# An interpretable machine learning model for predicting myocardial injury in patients with high cervical spinal cord injury

**DOI:** 10.3389/fgene.2025.1636065

**Published:** 2025-08-04

**Authors:** Jiaqi Li, Bingyu Zhang, Ye Liao, Liqin Wei, Qinfeng Huang, Lijun Lin, Jiaxin Chen, Hui Chen

**Affiliations:** ^1^ Department of Anesthesiology, Anesthesiology Research Institute, The First Affiliated Hospital, Fujian Medical University, Fuzhou, China; ^2^ Department of Anesthesiology, National Regional Medical Center Binhai Campus of the First Affiliated Hospital, Fujian Medical University, Fuzhou, China; ^3^ Department of Anesthesiology, Fujian Medical University Union Hospital, Fuzhou, China

**Keywords:** high cervical spinal cord injury, myocardial injury, machine learning, neural network, SHAP, risk prediction, interpretable artificial intelligence

## Abstract

**Background:**

High cervical spinal cord injury (HCSCI) is associated with severe autonomic dysfunction and an increased risk of cardiovascular complications, including myocardial injury. However, early identification of myocardial injury remains challenging because of the lack of predictive tools.

**Methods:**

A total of 454 patients with HCSCI were retrospectively enrolled and categorized into myocardial injury (n = 101) and non-injury (n = 353) groups. Univariate and multivariate logistic regression analyses were used to identify independent risk factors. Four machine learning (ML) models—logistic regression, gradient boosting machine (GBM), neural network (NeuralNetwork), and adaptive boosting (AdaBoost)—were constructed to predict myocardial injury, and model performance was evaluated using the area under the curve (AUC), F1 score, and average precision (AP). SHapley Additive exPlanations (SHAP) was applied for model interpretability.

**Results:**

Multivariate analysis identified dyspnea [odds ratio (OR) = 3.32; 95% confidence interval (CI): 1.49–7.39] and low hematocrit (OR = 2.18; 95% CI: 1.04–4.57) as independent predictors of myocardial injury. Among the ML models, the neural network model achieved the highest AUC and F1 score in the testing set and demonstrated superior calibration and net clinical benefit. The SHAP analysis revealed that dyspnea, low-density lipoprotein (LDL), spinal cord segment level, paralysis status, hematocrit, and myocardial injury stage were the top predictors. Individualized SHAP force plots illustrated the contribution of each feature to prediction outcomes.

**Conclusion:**

We developed an interpretable ML model capable of accurately predicting myocardial injury in patients with HCSCI. The neural network model showed the best overall performance and, with SHAP interpretation, provided transparent and individualized risk insights, supporting early diagnosis and targeted management in clinical practice.

## 1 Introduction

Traumatic spinal cord injury (SCI) remains a serious global health concern, with an estimated prevalence of 10.5 cases per 100,000 individuals (Kumar et al., 2018). SCI often results in permanent motor and sensory deficits below the level of injury, with substantial physical, psychological, and socioeconomic consequences. Inhospital mortality rates vary between 2.1% and 24.1%, influenced by injury severity, level, and access to specialized care (Chamberlain et al., 2015). Injuries at or above the T7 spinal segment are categorized as high-level SCI due to their complete interruption of sympathetic nervous system (SNS) function (Apstein et al., 1987; Xia et al., 2007), leading to profound autonomic dysregulation.

High-level SCI (HSCI) is frequently associated with multisystem complications during the acute phase, with cardiovascular dysfunction being a primary cause of early mortality (Grigorean et al., 2009; Partida et al., 2016). The loss of sympathetic tone and impaired autonomic regulation can lead to bradyarrhythmia, hypotension, reduced cardiac output, and myocardial ischemia, all of which increase the risks associated with surgery and anesthesia (Kumar et al., 2018; Chamberlain et al., 2015). Accumulating clinical evidence has highlighted the occurrence of early myocardial injury following high-level SCI (Chen et al., 2014). In experimental models, ultrastructural damage to cardiomyocytes has been observed shortly after injury, supporting the biological plausibility of this complication. Such cardiac changes can destabilize hemodynamics and predispose patients to perioperative circulatory collapse, representing a key challenge in early clinical management.

Machine learning (ML) has recently shown strong performance in predicting clinical outcomes and diagnoses. Compared with traditional statistical methods, ML algorithms are capable of handling complex interactions and nonlinear relationships, which makes them increasingly utilized in clinical research, where outcomes are often influenced by multifactorial and intricate dependencies (Stevens et al., 2020; Chung et al., 2023).

Despite significant and continuous advances in ML over the past decade, these models are often regarded as “black boxes” due to their lack of interpretability. Recent developments in interpretable artificial intelligence (AI) aim to address this limitation by enabling clinicians and research workers to understand how ML algorithms make decisions and generate outputs. Interpretable AI provides a set of methods that enhance the transparency of ML models, thus facilitating their translation into clinical practice (Saeed and Omlin, 2023).

Based on this, we hypothesize that ML models can assist clinicians in predicting myocardial injury in patients with HSCI using clinical data and that interpretable AI methods can further help clinicians explain model predictions to patients while deepening the understanding of the underlying risk factors contributing to myocardial injury.

The aim of this study is to develop an interpretable machine learning model using clinical data to predict myocardial injury during the treatment course of patients with high-level spinal cord injury and to evaluate the feasibility of applying interpretable ML models in clinical practice.

## 2 Methods

### 2.1 Study design and participants

Patients with HSCI who underwent elective surgery at the First Affiliated Hospital of Fujian Medical University between 1 June 2015 and 31 December 2022 were included in this retrospective study. All included patients had injuries at or above the T7 spinal segment. The level of injury was determined based on neurological examinations and imaging assessments. According to the American Spinal Injury Association (ASIA) Impairment Scale (AIS), all patients exhibited complete loss of motor and sensory function below the level of injury and were classified as ASIA grade (Ditunno et al., 1994).

A total of 454 patients were enrolled, including 359 male (79.1%) and 95 female (20.9%) patients, with ages ranging from 18 to 80 years. Based on the presence or absence of myocardial injury, patients were categorized into two groups: the myocardial injury group (n = 101) and the non-myocardial injury group (n = 353).

### 2.2 Diagnostic criteria for myocardial injury

The diagnosis of myocardial injury was established if any of the following criteria were met:

1. ST-segment elevation or T-wave inversion observed in two consecutive leads on electrocardiogram (ECG);
2. creatine kinase–MB isoenzyme (CK-MB) > 25 U/L, elevation of at least one myocardial enzyme (including CK-MB) accompanied by potential ECG abnormalities, or elevation of any myocardial enzyme combined with definitive ECG abnormalities, with ST-T changes being the most common manifestation;
3. cardiac troponin T (cTnT) > 0.5 μg/mL.

In the current study, myocardial injury was identified based on elevated preoperative plasma myocardial enzymes following spinal cord injury, including creatine kinase (CK), cardiac troponin T (cTnT), creatine kinase–MB isoenzyme (CK-MB), lactate dehydrogenase (LDH), and aspartate aminotransferase (AST) (Chen et al., 2014). Serum enzyme concentrations were monitored within 48 h after admission and again within 48 h postoperatively.

### 2.3 Observational indicators

Observational indicators were as follows: demographic characteristics, which included age (at admission) and sex; comorbidities, including the presence of arrhythmia, hypertension, diabetes, and other comorbid conditions; vital sign-related indicators, including admission hypotension, which is defined as systolic blood pressure <90 mmHg upon hospital admission, preoperative hypotension, which is defined as sustained systolic blood pressure <90 mmHg for more than 30 min prior to surgery, and dyspnea, which is defined as subjective complaints of shortness of breath or labored breathing during routine activities; SCI-related indicators, which included segments of spinal cord involved, which is classified as C1–C7 or T1–T7 injured segments, stage of high spinal cord injury, including acute and subacute phases, and paralysis status, including the presence or absence of complete paralysis; and laboratory parameters (measured within 24 h of admission), including hemoglobin (g/L), hematocrit (%), total cholesterol (TC, mmol/L), low-density lipoprotein (LDL, mmol/L), and serum albumin (g/L).

These parameters were extracted from electronic medical records, clinical progress notes, imaging and laboratory reports, physician orders, nursing documentation, anesthesia records, and recovery room monitoring sheets.

All the above variables were included while performing a univariate logistic regression analysis, and those with statistical significance (p < 0.05) were subsequently incorporated into multivariate regression and machine learning model construction.

### 2.4 Inclusion and evaluation criteria

Inclusion criteria were as follows:

(1) patients with HSCI who were discharged between 1 June 2015 and 31 December 2022 and whose symptom onset occurred within 2 weeks prior to admission;
(2) fulfillment of diagnostic criteria for myocardial injury;
(3) HSCI confirmed through imaging (MRI or CT) as trauma-related and who subsequently underwent surgical intervention for spinal cord injury;
(4) no history of preexisting cardiac disease and a baseline cardiac function classified as New York Heart Association (NYHA) class I–II;
(5) age between 18 and 80 years.

Exclusion criteria included the following:

(1) patients with impaired consciousness or diagnosed psychiatric disorders;
(2) SCI resulting from nontraumatic causes such as myelitis or iatrogenic injury, or from trauma mechanisms other than motor vehicle accidents, falls, or blunt force trauma;
(3) presence of preexisting cardiac disease, neuromuscular disorders, or severe hepatic or renal insufficiency prior to injury;
(4) incomplete or missing clinical data on any of the defined observational indicators.

### 2.5 Machine learning model

The univariate logistic regression analysis was first performed to identify potential risk factors significantly associated with myocardial injury. Variables with statistical significance (p < 0.05) were subsequently entered into a multivariate logistic regression model to determine independent predictors. Odds ratios (ORs) and 95% confidence intervals (CIs) were calculated for each variable.

We constructed and evaluated four supervised machine learning models: logistic regression, gradient boosting machine (GBM), neural network (NeuralNetwork), and adaptive boosting (AdaBoost). All models were implemented using the Python Scikit-learn library (version 1.0.1, https://github.com/scikit-learn/scikit-learn) (Tanaka, 2023). The dataset was randomly split into training (70%) and validation (30%) subsets. The bootstrap method was applied with 500 repetitions to derive the confidence intervals for area under the receiver operating characteristic curve (AUC), sensitivity, specificity, precision, and F1 score. AUC and F1 score were used as the primary metrics for model performance evaluation (Sokolova and Lapalme, 2009). All models were constructed using default hyperparameter settings in the Scikit-learn package.

To assess clinical utility, a decision curve analysis (DCA) was performed. Calibration curves were used to evaluate the agreement between predicted probabilities and actual outcomes. Additionally, precision–recall (PR) curves were plotted to assess performance under class imbalance conditions.

### 2.6 SHAP model interpretation

We applied SHapley Additive exPlanations (SHAP) to interpret the ML models and determine the contribution of each feature to the prediction. SHAP is a unified framework that enables both cohort-level and individual-level interpretation of ML model outputs (Lundberg and Lee, 2017).

For each feature, SHAP values were calculated for all patients, and the mean absolute SHAP values were aggregated to assess the global importance of each variable. The SHAP feature importance plot illustrates the overall contribution of each feature to the model’s predictions—features with larger mean absolute SHAP values are considered more influential. The SHAP summary plot provides a visualization of how each feature impacts the model output. Each dot in the summary plot represents the SHAP value of a specific feature for an individual patient, with color ranging from red to blue to represent high to low feature values, respectively. In addition, SHAP dependence plots were generated to explore how specific features influence model predictions and to assess potential interactions between variables. All SHAP analyses were performed in Python using the SHAP package (version 0.40.0).

### 2.7 Statistical analysis

Continuous variables were expressed as mean ± standard deviation or median with interquartile range (IQR), whereas categorical variables were presented as frequencies and percentages. Comparisons between categorical variables were performed using the Chi-square test, and comparisons of continuous variables between groups were conducted using the independent sample t-test. Paired t-tests were applied to assess changes in body composition and inflammatory markers. All statistical analyses were conducted using Python (version 3.7.3) and R statistical software (version 4.0.1). A two-tailed p-value <0.05 was considered statistically significant.

## 3 Result

### 3.1 General clinical data

**FIGURE 1:**
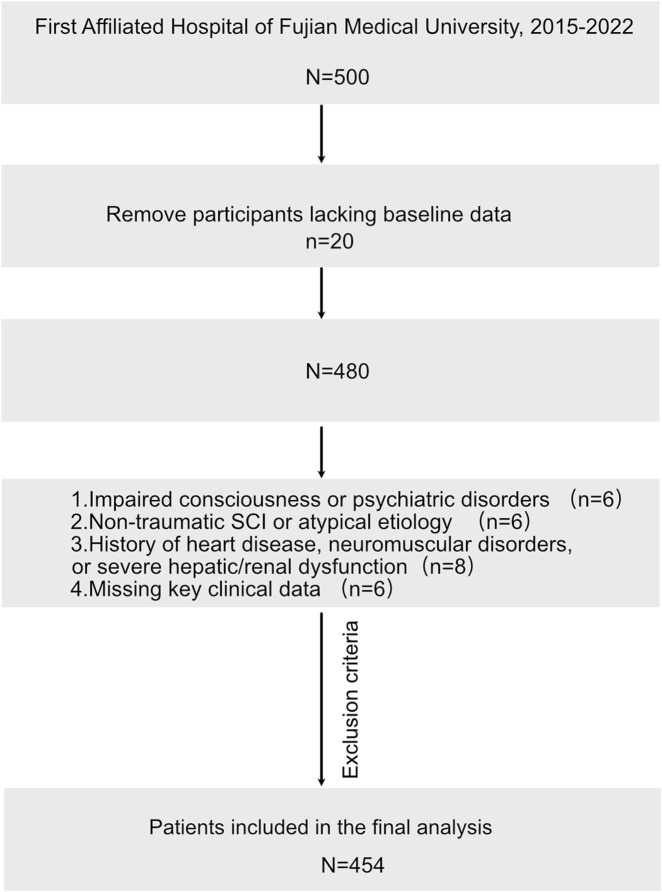
Flowchart of patient enrollment.

According to the inclusion criteria, a total of 454 patients with high cervical spinal cord injury (HCSCI) were identified (see flowchart, Figure 1); among them, 101 patients had myocardial injury and 353 patients did not. Significant differences were observed between the two groups in sex, admission to hospital with hypotension, preoperative hypotension, dyspnea, segments of spinal cord affected, other comorbidities, paralysis, stage of high spinal cord injury, hemoglobin, hematocrit, TC, LDL, and albumin (p < 0.05) (Table 1).

**TABLE 1:** Characteristics of myocardial injury in patients with high spinal cord injury.

Characteristics	Myocardial damage		P
	No	Yes	
N	353	101	
Age (years), n (%)			1
<65	267 (75.6)	77 (76.2)	
≥65	86 (24.4)	24 (23.8)	
Sex, n (%)			0.034
Female	82 (23.2)	13 (12.9)	
Male	271 (76.8)	88 (87.1)	
Arrhythmia, n (%)			0.452
No	239 (67.7)	73 (72.3)	
Yes	114 (32.3)	28 (27.7)	
Hypertension, n (%)			0.164
No	280 (79.3)	87 (86.1)	
Yes	73 (20.7)	14 (13.9)	
Diabetes, n (%)			0.426
No	321 (90.9)	95 (94.1)	
Yes	32 (9.1)	6 (5.9)	
Admission to hospital with hypotension, n (%)			0.019
No	297 (84.1)	74 (73.3)	
Yes	56 (15.9)	27 (26.7)	
Preoperative hypotension, n (%)			0.016
No	327 (92.6)	85 (84.2)	
Yes	26 (7.4)	16 (15.8)	
Dyspnea, n (%)			<0.001
No	319 (90.4)	68 (67.3)	
Yes	34 (9.6)	33 (32.7)	
Segments of spinal cord, n (%)			<0.001
T1–T6	41 (11.6)	36 (35.6)	
C1–C7	312 (88.4)	65 (64.4)	
Other comorbidities, n (%)			<0.001
No	292 (82.7)	64 (63.4)	
Yes	61 (17.3)	37 (36.6)	
The stage of high spinal cord injury, n (%)			<0.001
Subacute phase	180 (51.0)	23 (22.8)	
Acute phase	173 (49.0)	78 (77.2)	
Paralysis, n (%)			<0.001
Incomplete paraplegia	53 (15.0)	36 (35.6)	
Complete paralysis	300 (85.0)	65 (64.4)	
Hemoglobin (g/mL), n (%)			<0.001
≥110	314 (89.0)	76 (75.2)	
<110	39 (11.0)	25 (24.8)	
Hematocrit, n (%)			<0.001
>0.387	197 (55.8)	29 (28.7)	
≤0.387	156 (44.2)	72 (71.3)	
TC [(mean (SD)]	4.52 (1.01)	3.80 (0.89)	<0.001
LDL, n (%)			<0.001
>2.8	204 (57.8)	23 (22.8)	
≤2.8	149 (42.2)	78 (77.2)	
Albumin [(mean (SD)]	37.62 (4.22)	35.67 (4.79)	<0.001

The cohort data were divided into training and testing sets at a 7:3 ratio, with baseline data for the training and testing sets shown in Table 2. Except for preoperative hypotension and segments of spinal cord affected, where significant differences were observed between the training and testing sets (p < 0.05), no significant differences were found between the training and testing groups for other baseline variables (p > 0.05).

**TABLE 2:** Clinical characteristics of patients in training and testing sets.

Variable	Training set (n = 319)	Testing set (n = 135)	p
TC [mean (SD)]	4.41 (1.00)	4.25 (1.08)	0.119
LDL [mean (SD)]	2.89 (0.99)	2.83 (1.07)	0.601
Albumin [mean (SD)]	37.19 (4.28)	37.16 (4.77)	0.938
Myocardial injury, n (%)			1
No	248 (77.7)	105 (77.8)	
Yes	71 (22.3)	30 (22.2)	
Age (years), n (%)			0.442
<65	238 (74.6)	106 (78.5)	
≥65	81 (25.4)	29 (21.5)	
Sex, n (%)			0.283
Female	62 (19.4)	33 (24.4)	
Male	257 (80.6)	102 (75.6)	
Arrhythmia, n (%)			0.344
No	224 (70.2)	88 (65.2)	
Yes	95 (29.8)	47 (34.8)	
Hypertension, n (%)			0.923
No	257 (80.6)	110 (81.5)	
Yes	62 (19.4)	25 (18.5)	
Diabetes, n (%)			1
No	292 (91.5)	124 (91.9)	
Yes	27 (8.5)	11 (8.1)	
Admission_to_hospital_with_hypotension, n (%)			0.629
No	263 (82.4)	108 (80.0)	
Yes	56 (17.6)	27 (20.0)	
Preoperative_hypotension, n (%)			0.033
No	296 (92.8)	116 (85.9)	
Yes	23 (7.2)	19 (14.1)	
Dyspnea, n (%)			0.3
No	276 (86.5)	111 (82.2)	
Yes	43 (13.5)	24 (17.8)	
Segments_of_spinal_cord, n (%)			0.019
T1–T7	45 (14.1)	32 (23.7)	
C1–C5	274 (85.9)	103 (76.3)	
Other_comorbidities, n (%)			0.402
No	254 (79.6)	102 (75.6)	
Yes	65 (20.4)	33 (24.4)	
The stage of high spinal cord injury, n (%)			0.7
Subacute phase	145 (45.5)	58 (43.0)	
Acute phase	174 (54.5)	77 (57.0)	
Paralysis, n (%)			0.119
Incomplete paraplegia	263 (82.4)	102 (75.6)	
Complete paralysis	56 (17.6)	33 (24.4)	
Hemoglobin (g/mL), n (%)			0.665
≥110	276 (86.5)	114 (84.4)	
<110	43 (13.5)	21 (15.6)	
Hematocrit, n (%)			0.169
>0.387	166 (52.0)	60 (44.4)	
≤0.387	153 (48.0)	75 (55.6)	

Myocardial injury, defined as elevated serum cardiac biomarkers (troponins, CK-MB) or electrocardiogram changes; hypertension, history of hypertension; diabetes, history of diabetes; preoperative hypotension, defined as systolic blood pressure <90 mmHg for more than 30 min before surgery; dyspnea, defined as shortness of breath or difficulty breathing during routine activities; segments of spinal cord affected, the number of spinal cord segments involved in the injury; other comorbidities, presence of additional comorbidities; acute phase (within 24 h after injury) and subacute phase (24 h–2 weeks after injury); paralysis, whether full paralysis (complete loss of motor function) is present; hemoglobin, blood hemoglobin level; abnormal if below 110 g/L; hematocrit, hematocrit level; TC, total cholesterol (in mmol/L); LDL, low-density lipoprotein (in mmol/L); albumin, serum albumin level (in g/L).

### 3.2 Screening and analysis of risk factors for myocardial injury in patients with HCSCI

Univariate analysis identified several significant risk factors for myocardial injury in patients with HCSCI, including male sex (OR = 2.61; 95% CI: 1.13–6.01; p = 0.025), preoperative hypotension (OR = 2.43; 95% CI: 1.00–5.87; p = 0.049), dyspnea (OR = 4.31; 95% CI: 2.20–8.45; p < 0.001), C1–C5 of multiple spinal cord segments (OR = 0.26; 95% CI: 0.13–0.49; p < 0.001), presence of other comorbidities (OR = 2.14; 95% CI: 1.17–3.90; p = 0.013), acute phase of high spinal cord injury (OR = 3.09; 95% CI: 1.71–5.57; p < 0.001), complete paralysis (OR = 3.12; 95% CI: 1.68–5.79; p < 0.001), low hemoglobin levels (OR = 2.69; 95% CI: 1.36–5.30; p = 0.004), low hematocrit (OR = 2.62; 95% CI: 1.51–4.56; p < 0.001), decreased TC (OR = 0.45; 95% CI: 0.32–0.63; p < 0.001), LDL (OR = 0.42; 95% CI: 0.30–0.60; p < 0.001), and serum albumin (OR = 0.92; 95% CI: 0.86–0.98; p = 0.008) (Table 3).

**TABLE 3:** Results of logistic regression analysis of myocardial injury in patients with high spinal cord injury in training sets.

Variable	Univariate	P	Multivariate	P
	OR (95% CI)		OR (95% CI)	
Age (years), %				
<65	1.00 [reference]	p = 0.993		
≥65	1.00 (0.54–1.83)			
Sex (%)		p = 0.025		p = 0.004
Female	1.00 [reference]			
Male	2.61 (1.13–6.01)		4.27 (1.58–11.52)	
Arrhythmia (%)		p = 0.528		
No	1.00 [reference]			
Yes	0.83 (0.46–1.49)			
Hypertension (%)		p = 0.107		
No	1.00 [reference]			
Yes	0.53 (0.25–1.14)			
Diabetes (%)		p = 0.070		
No	1.00 [reference]			
Yes	0.26 (0.06–1.12)			
Admission_to_hospital_with_hypotension (%)		p = 0.111		
No	1.00 [reference]			
Yes	1.69 (0.89–3.21)			
Preoperative_hypotension (%)		p = 0.049		p = 0.718
No	1.00 [reference]		1.00 [reference]	
Yes	2.43 (1.00–5.87)		1.21 (0.43–3.45)	
Dyspnea (%)		p < 0.001		p = 0.003
No	1.00 [reference]		1.00 [reference]	
Yes	4.31 (2.20–8.45)		3.32 (1.49–7.39)	
Segments_of_spinal_cord (%)		p < 0.001		p = 0.021
T1–T7	1.00 [reference]		1.00 [reference]	
C1–C5	0.26 (0.13–0.49)		0.39 (0.18–0.87)	
Other_comorbidities (%)		p = 0.013		p = 0.961
No	1.00 [reference]		1.00 [reference]	
Yes	2.14 (1.17–3.90)		0.98 (0.44–2.18)	
The stage of high spinal cord injury (%)		p < 0.001		p = 0.005
Subacute phase	1.00 [reference]		1.00 [reference]	
Acute phase	3.09 (1.71–5.57)		2.68 (1.34–5.35)	
Paralysis (%)		p < 0.001		p = 0.146
Incomplete paraplegia	1.00 [reference]		1.00 [reference]	
complete paralysis	3.12 (1.68–5.79)		1.74 (0.82–3.67)	
Hemoglobin (g/mL), %		p = 0.004		p = 0.346
≥110	1.00 [reference]		1.00 [reference]	
<110	2.69 (1.36–5.30)		1.55 (0.62–3.83)	
Hematocrit (%)		p < 0.001		p = 0.038
>0.387	1.00 [reference]		1.00 [reference]	
≤0.387	2.62 (1.51–4.56)		2.18 (1.04–4.57)	
TC [mean (SD)]	0.45 (0.32–0.63)	p < 0.001	1.24 (0.59–2.64)	p = 0.572
LDL [mean (SD)]	0.42 (0.30–0.60)	p < 0.001	0.54 (0.26–1.10)	p = 0.089
Albumin [mean (SD)]	0.92 (0.86–0.98)	p = 0.008	1.03 (0.95–1.13)	p = 0.443

Multivariate logistic regression analysis demonstrated that dyspnea (OR = 3.32; 95% CI: 1.49–7.39; p = 0.003) and low hematocrit (OR = 2.18; 95% CI: 1.04–4.57; p = 0.038) were independently associated with myocardial injury in patients with HCSCI (Table 3). Variables that showed statistical significance in the univariate analysis were included in the subsequent model construction.

### 3.3 Integrated analysis of multiple classification models

**FIGURE 2:**
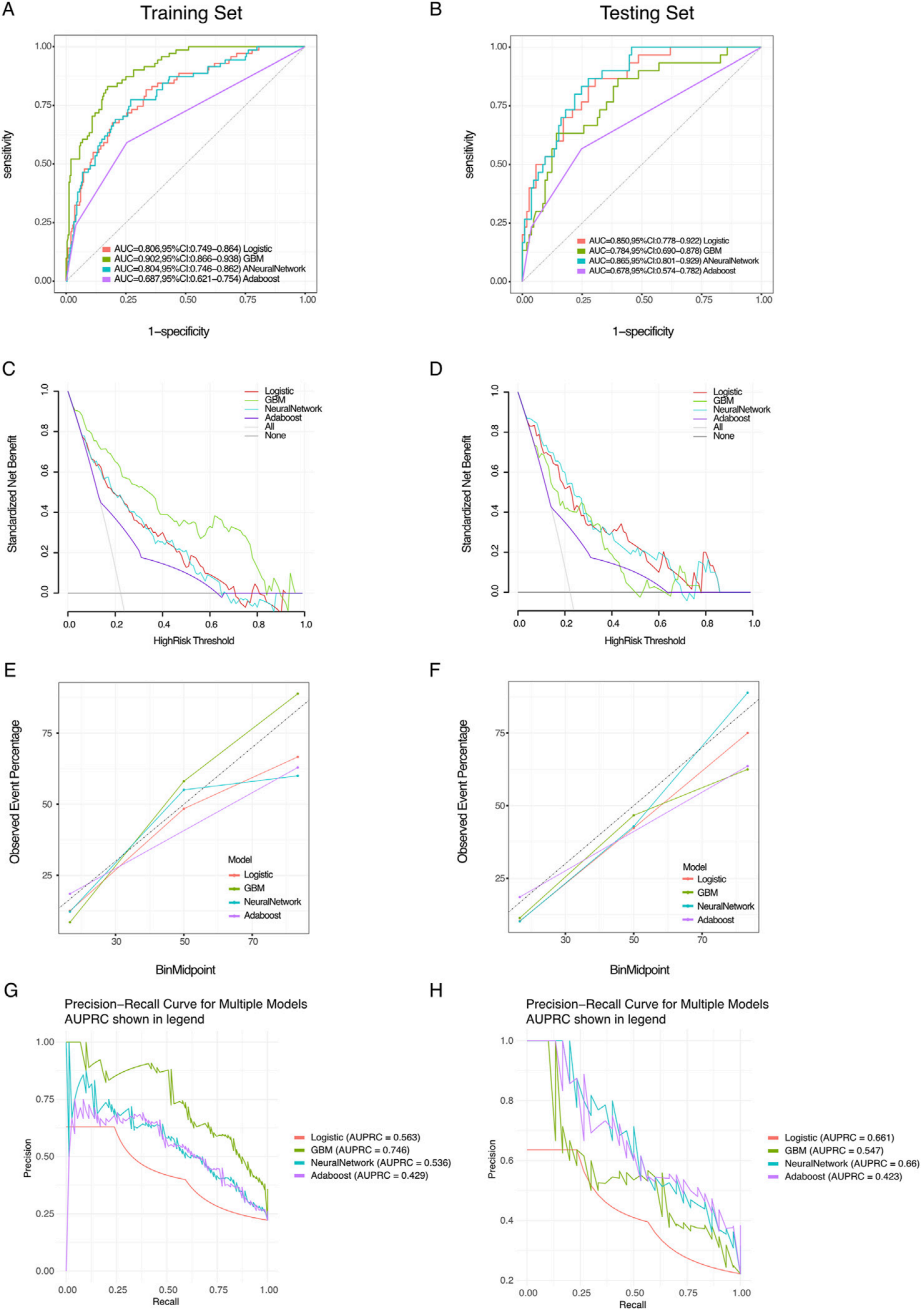
Performance evaluation of various models. **(A,B)** Receiver operating characteristic (ROC) curves for training and testing sets: the ROC curves demonstrate the performance of the logistic, GBM, NeuralNetwork, and Adaboost models. The area under the curve (AUC) values are indicated in the legend for both the training set **(A)** and testing set **(B)**. **(C,D)** Standardized misclassification rate: these plots display the standardized misclassification rate for each model as a function of the decision threshold for both the training **(C)** and testing sets **(D)**. **(E,F)** Observed event percentage vs. binned threshold: these plots show the observed event percentage against binned thresholds for the training **(E)** and testing sets **(F)**, providing insight into the calibration performance of each model. **(G,H)** Precision–recall curves for multiple models: the precision–recall curves for each model are presented, with the area under the precision–recall curve (AUPRC) shown in the legend for both the training **(G)** and testing sets **(H)**.

The logistic, GBM, NeuralNetwork, and AdaBoost models were each trained and repeated 10 times. Model performance was evaluated using the AUC metric (Obuchowski and Bullen, 2018). The results showed that GBM performed best on the training set, whereas NeuralNetwork achieved the highest AUC on the testing set (Figures 2A,B). However, AUC reflects only the discriminative ability of the model and does not indicate its clinical utility or practical superiority among different models (Obuchowski and Bullen, 2018; Muschelli, 2020). Therefore, we further analyzed decision curve analysis (DCA), calibration curves, and PR curves.

DCA results indicated that NeuralNetwork yielded greater clinical net benefit in the training set (Figure 2C). In contrast, in the testing set, GBM, NeuralNetwork, and AdaBoost demonstrated comparable clinical applicability (Figure 2D). Calibration curves showed similar performance among the four models in the training set (Figure 2E), whereas in the testing set, NeuralNetwork exhibited better alignment between predicted probabilities and observed outcomes (Figure 2F). In terms of average precision (AP), GBM performed best in the training set (Figure 2G), whereas NeuralNetwork outperformed other models in the testing set, with the highest AP score (Figure 2H).

The F1 score was used to further compare the performance of the machine learning models. As the F1 score provides a balanced measure of a model’s performance on imbalanced datasets—capturing the harmonic mean of precision (positive predictive value) and recall (sensitivity)—it is particularly informative in this context (Wang et al., 2021; Tack, 2019). In the training set, the NeuralNetwork model achieved the highest F1 score (Table 4), and it also outperformed other models in the testing set (Table 5). Taken together, these findings suggest that the NeuralNetwork model offers the most balanced and optimal predictive performance. Therefore, the NeuralNetwork model was selected for subsequent analysis.

**TABLE 4:** Evaluation of model performance in the training set.

Model	Threshold	Accuracy	Sensitivity	Specificity	Precision	F1
Logistic	0.267817194	0.777	0.676	0.806	0.5	0.575
GBM	0.24291048	0.828	0.831	0.827	0.578	0.682
NeuralNet	0.223332124	0.74	0.775	0.73	0.451	0.57
Adaboost	0.143939187	0.712	0.592	0.746	0.4	0.477

Model abbreviations: Logistic, logistic regression; GBM, gradient boosting machine; NeuralNet, neural network; Adaboost, adaptive boosting.

**TABLE 5:** Evaluation of model performance in the testing set.

Model	Threshold	Accuracy	Sensitivity	Specificity	Precision	F1
Logistic	0.237004354	0.733	0.867	0.695	0.448	0.591
GBM	0.354197911	0.807	0.633	0.857	0.559	0.594
NeuralNet	0.256997667	0.756	0.867	0.724	0.473	0.612
Adaboost	0.143939187	0.711	0.567	0.752	0.395	0.466

Model abbreviations: Logistic, logistic regression; GBM, gradient boosting machine; NeuralNet, neural network; Adaboost, adaptive boosting.

### 3.4 SHAP model interpretation

**FIGURE 3:**
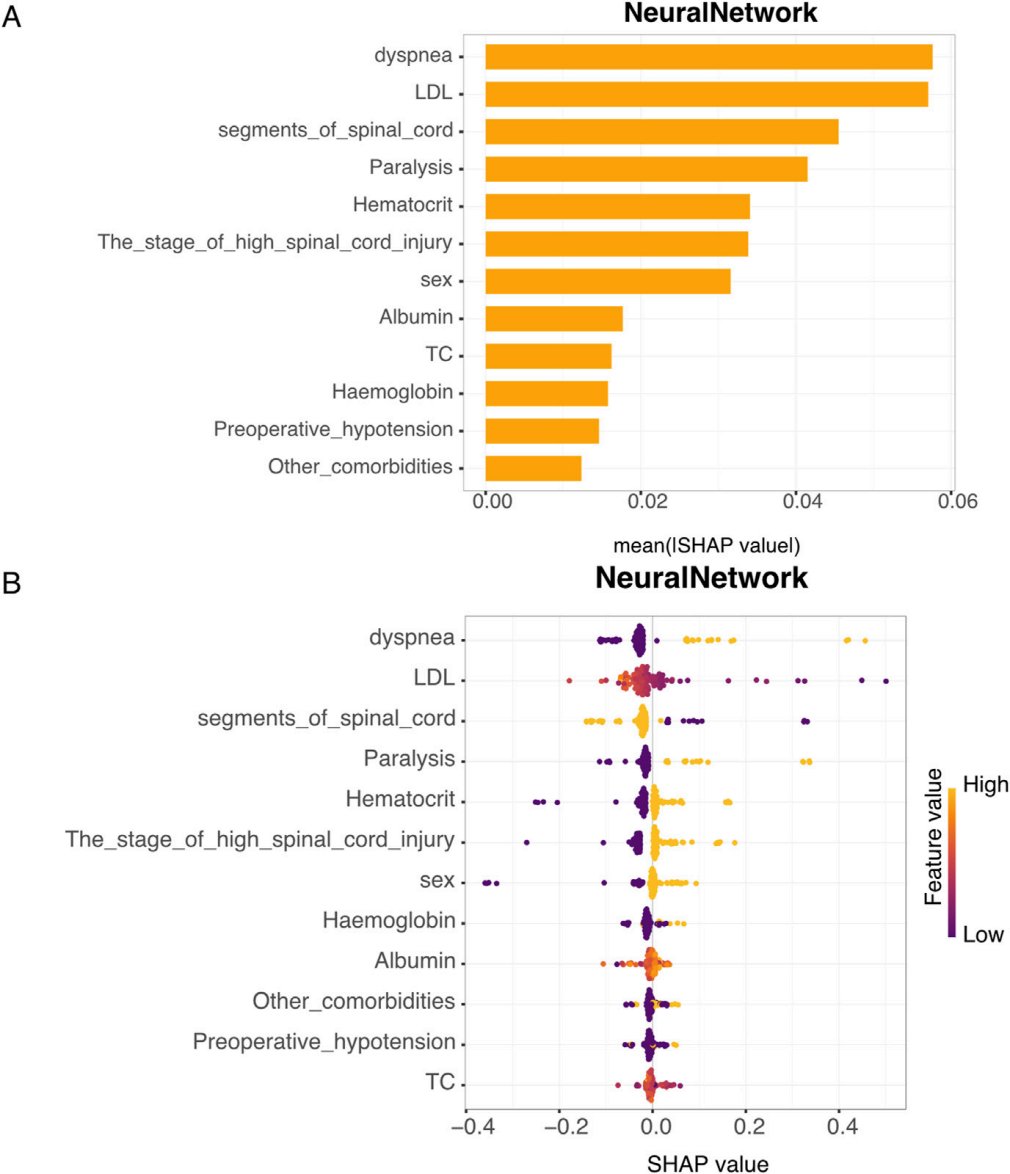
SHAP value analysis of the neural network model. **(A)** Feature importance: this bar plot displays the mean SHAP (SHapley Additive exPlanations) value of each feature, which quantifies the average contribution of each variable to the model’s predictions across all patients. The Y-axis lists the input features used in the neural network model (e.g., dyspnea, LDL, and spinal cord segment). The X-axis represents the average absolute SHAP value for each feature, which indicates its overall importance: higher values reflect greater impact on the model’s output. Features such as dyspnea, LDL, and segments_of_spinal_cord are among the most influential in predicting myocardial injury. **(B)** SHAP value distribution: this plot shows the distribution of SHAP values for each feature, with color indicating the level of the feature value. Higher feature values result in more significant SHAP values.

Figure 3A illustrates the SHAP-based feature importance ranking derived from the NeuralNetwork model. Features were ranked from the highest to lowest according to their mean absolute SHAP values, reflecting their relative contributions to the model’s predictive outcomes. The top six influential features were, namely, dyspnea, LDL, the number of spinal cord segments involved, paralysis status, hematocrit level, and the stage of HCSCI.

Figure 3B presents the SHAP summary plot from the NeuralNetwork model, highlighting the individual contributions of each feature to the prediction. A higher SHAP value indicates a stronger positive impact on predicting myocardial injury. For example, patients with dyspnea were more likely to be predicted as having myocardial injury than those without dyspnea. Patients with injury levels located at T1–T7 were at higher predicted risk than those with injuries at C1–C5. Additionally, lower LDL levels were associated with an increased predicted risk of myocardial injury. Similarly, elevated hematocrit was also linked to a higher likelihood of myocardial injury.

In addition, the study presents two individual SHAP explanation plots. In these visualizations, the color indicates the direction of each feature’s contribution to the model prediction—red denotes a positive contribution, and blue denotes a negative contribution—whereas the length of the color bar reflects the magnitude of the contribution.

**FIGURE 4:**
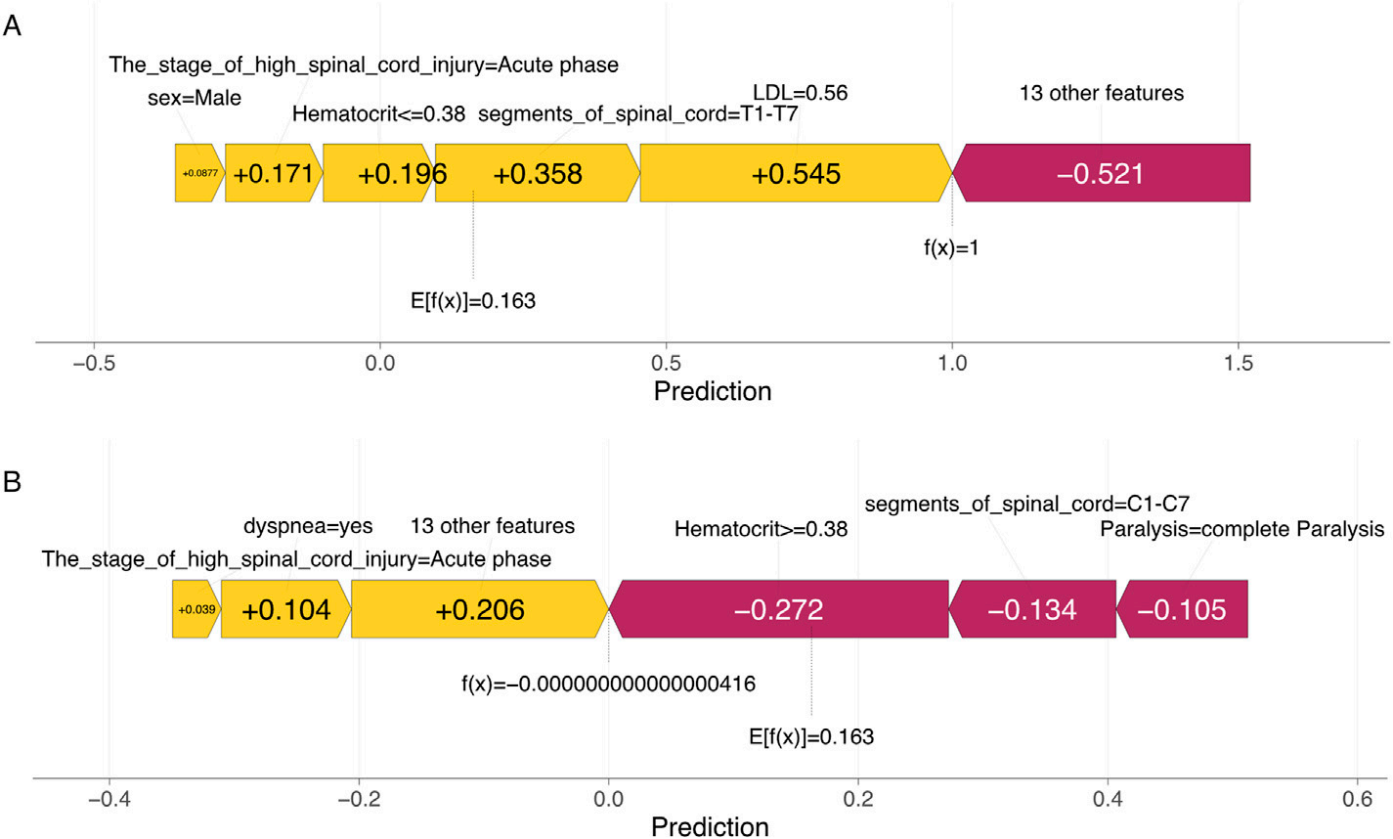
Explanation of machine learning model at the patient level. **(A)** SHAP force plot illustrating the prediction of myocardial injury in patient A with HCSCI. **(B)** SHAP force plot illustrating the prediction of the absence of myocardial injury in patient B with HCSCI.

For patient A in the true positive group, the SHAP plot generated by the NeuralNetwork model revealed that being female, in the acute phase of high spinal cord injury, elevated hematocrit levels, an injury level located at T1–T7, and an LDL value of 0.56 collectively contributed to the prediction of myocardial injury (Figure 4A).

For patient B in the true negative group, the SHAP plot indicated that reduced hematocrit, an injury level at C1–C5, and complete paralysis were associated with the absence of myocardial injury (Figure 4B).

## 4 Discussion

In this study, we systematically analyzed the risk factors for myocardial injury in patients with HCSCI and developed a predictive model using multiple ML algorithms. The findings highlight both clinical risk indicators and the interpretability of model outputs, offering valuable insights for individualized risk stratification and early intervention in this vulnerable patient population.

From a clinical perspective, univariate and multivariate analyses revealed that dyspnea and low hematocrit were independently associated with an increased risk of myocardial injury. These findings align with previous studies, indicating that respiratory compromise (Berlowitz et al., 2016) and anemia (Fowler et al., 2015) are common complications following cervical spinal cord injury and are strongly associated with cardiovascular instability. Dyspnea reflects impaired respiratory muscle function, which may increase cardiac workload and reduce oxygen delivery, thereby aggravating myocardial vulnerability (Eldahan and Rabchevsky, 2018). Likewise, low hematocrit (Fowler et al., 2015) levels may indicate reduced oxygen-carrying capacity, exacerbating tissue hypoxia and increasing the likelihood of myocardial injury. These physiological mechanisms provide a reasonable explanation for the observed statistical associations.

In addition to these independent predictors, several other variables were significantly associated with myocardial injury in the univariate analysis, including male sex, preoperative hypotension, complete paralysis, low serum albumin, and dyslipidemia (i.e., low TC and LDL levels) (Craggs, 2006). Interestingly, patients with injury segments located at T1–T7 had a higher predicted risk of myocardial injury than those with injuries at the C1–C7 level. This could reflect differences in sympathetic nervous system involvement and hemodynamic regulation at varying spinal levels (Teasell et al., 2000). These findings reinforce the multifactorial nature of myocardial injury in HCSCI and support a comprehensive approach to early risk screening.

In the modeling phase, four classification algorithms—logistic regression, GBM, NeuralNetwork, and AdaBoost—were evaluated. The NeuralNetwork model demonstrated the most robust and consistent performance across multiple metrics, including AUC, AP, F1 score, and calibration. Although GBM showed the highest AUC in the training set, the NeuralNetwork model outperformed all others in the testing set, particularly in terms of the F1 score and AP, which are critical for evaluating imbalanced datasets. The DCA further indicated that the NeuralNetwork model offered better clinical net benefit, especially in the training set, suggesting its potential utility in real-world clinical decision-making.

A key strength of this study is the interpretability of the machine learning model using SHAP values. SHAP analysis not only ranked feature importance but also revealed how each variable influenced individual predictions. Dyspnea, LDL, number of spinal cord segments involved, paralysis status, hematocrit, and injury stage were identified as the most influential predictors in the NeuralNetwork model. The visualization of SHAP summary plots and patient-specific force plots further enhances the transparency of the model, facilitating clinician understanding and trust in the prediction outputs. For example, patient A’s SHAP force plot revealed that factors such as acute injury phase, elevated hematocrit, and low LDL collectively contributed to the positive prediction of myocardial injury. Conversely, in patient B, the absence of myocardial injury was associated with reduced hematocrit, cervical-level injury (C1–C5), and complete paralysis. These individualized insights demonstrate the potential of SHAP to support precision medicine in complex neurological populations.

The integration of machine learning with interpretable methods like SHAP addresses a major limitation in traditional clinical prediction models—the lack of transparency. By bridging predictive accuracy with clinical interpretability, our study offers a practical tool that can be translated into bedside use. Furthermore, the inclusion of both clinical variables and laboratory indices ensures that the model captures a holistic picture of patient status.

However, several limitations should be acknowledged. First, this was a single-center retrospective study, which may introduce selection bias and limit generalizability. External validation using multicenter datasets is necessary to confirm the robustness of the findings. Second, although the NeuralNetwork model showed strong performance, deep learning models require larger sample sizes to achieve their full predictive potential. Future work should explore ensemble models and incorporate temporal data to enhance prediction in real-time clinical settings.

## 5 Conclusion

In conclusion, in this study, we identify key clinical and laboratory predictors of myocardial injury in patients with HCSCI and propose an interpretable machine learning model with strong predictive performance. The NeuralNetwork model, supported by SHAP analysis, not only improves the accuracy but also enhances the understanding of individual risk profiles, providing a promising tool for early warning and personalized management in spinal cord injury care.

## Data Availability

The original contributions presented in the study are included in the article/Supplementary Material; further inquiries can be directed to the corresponding author.

## Abbreviations

AdaBoost, adaptive boosting; AUC, area under the receiver operating characteristic curve; AP, average precision; AST, aspartate transaminase; CK, creatine kinase; CK-MB, creatine kinase isoenzyme MB; DCA, decision curve analysis; F1, F1 score (harmonic mean of precision and recall); GBM, gradient boosting machine; HCSCI, high cervical spinal cord injury; LDH, lactate dehydrogenase; LDL, low-density lipoprotein; ML, machine learning; NeuralNetwork, neural network; PR curve, precision–recall curve; RF, random forest; ROC curve, receiver operating characteristic curve; SHAP, SHapley Additive exPlanations; TC, total cholesterol.
